# Salt and oxidative stresses uniquely regulate tomato cytokinin levels and transcriptomic response

**DOI:** 10.1002/pld3.71

**Published:** 2018-07-19

**Authors:** Erika A. Keshishian, H. Tucker Hallmark, Thiruvarangan Ramaraj, Lenka Plačková, Anitha Sundararajan, Faye Schilkey, Ondřej Novák, Aaron M. Rashotte

**Affiliations:** ^1^ Department of Biological Sciences Auburn University Auburn Alabama; ^2^ National Center for Genome Resources Santa Fe New Mexico; ^3^ Laboratory of Growth Regulators Centre of the Region Haná for Biotechnological and Agricultural Research Faculty of Science of Palacký University & Institute of Experimental Botany of the Czech Academy of Sciences Olomouc Czech Republic

**Keywords:** abiotic stress, cytokinin, oxidative stress, RNA‐sequencing, salt, *Solanum lycopersicum*, tomato, transcriptome

## Abstract

Cytokinins are well‐known to be involved in processes responsible for plant growth and development. More recently, these hormones have begun to be associated with stress responses as well. However, it is unclear how changes in cytokinin biosynthesis, signaling, or transport relate to stress effects. This study examines in parallel how two different stresses, salt, and oxidative stress, affect changes in both cytokinin levels and whole plant transcriptome response. *Solanum lycopersicum* seedlings were given a short‐term (6 hr) exposure to either salt (150 mM NaCl) or oxidative (20 mM H_2_O_2_) stress and then examined to determine both changes in cytokinin levels and transcriptome. LC‐MS/MS was used to determine the levels of 22 different types of cytokinins in tomato plants including precursors, active, transported, and conjugated forms. When examining cytokinin levels we found that salt treatment caused an increase in both active and inactive cytokinin levels and oxidative stress caused a decrease in these levels. RNA‐sequencing analyses of these same stress‐treated tissues revealed 6,643 significantly differentially expressed genes (DEGs). Although many DEGs are similar between the two stresses, approximately one‐third of the DEGs in each treatment were unique to that stress. Several cytokinin‐related genes were among the DEGs. Examination of photosystem II efficiency revealed that cytokinins affect physiological response to stress in tomato, further validating the changes in cytokinin levels seen *in planta*.

## 1 INTRODUCTION

Abiotic stress results in billions of dollars of crop losses every year, including in the important crop plant tomato (Acosta‐Motos et al., 2017; Pineda et al., 2012; Qin, Shinozaki, & Yamaguchi‐Shinozaki, 2011). Tomato growth and production is highly dependent on healthy functioning and photosynthesizing plants that are resistant to or tolerant of abiotic environmental stresses. Two primary stresses affecting tomato are salt stress and the broad stress of oxidative damage (Acosta‐Motos et al., 2017; Pineda et al., 2012). These stresses are often thought of as overlapping in their effects, yet are also thought to be distinct in mode of action. In tomato, salt stress has been examined by a number of researchers, while oxidative stress is virtually unstudied (Acosta‐Motos et al., 2017).

Salt stress‐related crop losses are a problem growing in magnitude as salt levels in farm lands are increasing due to abundant irrigation that continues to add residual salt. In tomatoes, salt stress is well known to reduce plant size, fresh weight, and photosynthesis (as recently reviewed in Acosta‐Motos et al., 2017). As such attempts to understand how plants respond to salt stress to generate resistant or tolerant lines is of great importance. Salt stress is known to affect plants in two basic ways: either through osmotic complications or through ion toxicity (Acosta‐Motos et al., 2017; Ismail & Horie, 2017). However, what remains unclear are mechanistic actions that occur as a plant responds to salt stress from either or both of these effects, particularly in regard to the genes involved in salt tolerance and resistance. Although there has been a great deal of progress made in understanding some of the basic components, signaling pathways, and initial genes involved in plant salt stress response, there is still much that is unknown (Acosta‐Motos et al., 2017; Ismail & Horie, 2017; Singh, Singh, Prasad, & Singh, 2017).

Oxidative stress produced during abiotic stresses is highly detrimental to growth at all stages of development and can greatly reduce crop yields in plants (as reviewed in Pandey et al., 2011; Qin et al., 2011; Duque et al., 2013). In tomato, oxidative stress is a major abiotic factor (Pandey et al., 2011), which causes damage at both tissue and cellular levels that can rupture membranes, breakdown photosynthetic machinery, and cause cell death (Zwack & Rashotte, 2015). Plants utilize the redox potential of molecular oxygen (O_2_) and water (H_2_O) to drive both reductive (photosynthesis) and oxidative (respiration) energy production. During each process, partially reduced/oxidized O_2_/H_2_O can form. Such redox intermediates readily oxidize other molecules and are known as reactive oxygen species (ROS) (Apel & Hirt, 2004; Gill & Tuteja, 2010). ROS or oxidative stress damage can occur during various abiotic stresses, such as extreme temperatures that alter membrane fluidity disrupting electron transport chain reactions (Mittler, Finka, & Goloubinoff, 2012) and high light levels that overwhelm photosystem II and its light‐harvesting complexes with excitation energy (Gill & Tuteja, 2010). ROS can additionally be produced directly through O_2_ interactions with heavy metals, UV radiation and pollutants such as ozone and sulfur dioxide (Gill & Tuteja, 2010; Mittler, 2002). As many stresses stimulate a response that includes the production of ROS, it is often involved in general stress responses (Apel & Hirt, 2004; Mittler, Vanderauwera, Gollery, & Van Breusegem, 2004; Saxena, Srikanth, & Chen, 2016). Yet for some crop species such as tomato, only a handful of genes have been linked to oxidative stress and require additional study.

An underexplored avenue for regulation of both salt and oxidative stress responses is the plant hormone cytokinin. Cytokinins are essential to plant hormones involved in numerous plant growth and developmental processes of great agronomic importance (Mok & Mok, 2001; Davies, 2004; To & Kieber, 2008; Muller, 2011). Notably, while both salt and oxidative stresses are detrimental to plant health and linked to abiotic stress senescence, cytokinins are connected to delaying plant senescence. Cytokinins have also been strongly linked to plant abiotic stress responses in studies examining cytokinin biosynthesis, metabolism, and signaling, where changes in cytokinin levels, responsive genes, or receptor mutants have dramatically altered growth under stress conditions (as seen or reviewed in Peleg & Blumwald, 2011; Wilkinson, Kudoyarova, Veselov, Arkhipova, & Davies, 2012; Nishiyama et al., 2011; Ramireddy, Chang, & Schmülling, 2014; Zwack & Rashotte, 2015; Veselov, Kudoyarova, Kudryakova, & Kusnetsov, 2017). This has also been shown in tomato for salt stress, albeit to a lesser degree (Ghanem et al., 2008, 2011; Pandey et al., 2011; Pineda et al., 2012; Žižková et al., 2015). Oxidative stress remains largely unstudied in tomato (Bose, Rodrigo‐Moreno, & Shabala, 2014; Mittova, Tal, Volokita, & Guy, 2003). As such, little is known in tomato of the mechanisms behind or pertinent genes involved in cytokinin‐based regulation of salt or oxidative stress.

This study examines connections between salt stress, oxidative stress, and cytokinins. Distinct changes in cytokinin levels after salt and oxidative stress along with distinct transcriptome changes were found. Specific alterations were identified in cytokinin‐related genes under each stress condition. These distinctive changes could be further linked to the efficiency of photosystem II (*F*_v_/*F*_m_) under salt and oxidative stress conditions. Furthermore, cytokinins have effects on *F*_v_/*F*_m_ under these stress conditions, possibly connecting the changes in cytokinin levels *in planta* to the observed physiological stress responses.

## 2 MATERIALS AND METHODS

### 2.1 Tomato seedling growth and stress exposure

*Solanum lycopersicum* cv. MicroTom seeds were planted in damp sunshine #8 soil mix, grown in 16 hr, 26°C light and 8 hr, 22°C dark periods with light supplemented at 150 μE m^−2^ s^−1^. For stress treatments seedlings, 10 days after germination were excised from soil with roots intact and soil was carefully removed. Initial examinations of tomato pTCS::VENUS lines, a generous gift from Dr. Yuval Eshed, were made after seedlings were exposed to one of four treatments for 24 hr under gentle shaking conditions at room temperature: MES + 5 μM *trans*‐zeatin, MES + 150 mM NaCl, MES + 20 mM H_2_O_2_, or MES buffer (3 mM MES buffer, pH 5.7) as a control. Roots from three plants in three biological treated replicates were viewed using a Nikon Eclipse 80i microscope epifluorescence microscope with a UV source and a yellow fluorescent protein (YFP) filter. A representative photo of a root from each treatment was taken with a Qimaging Fast 1394 digital camera and cropped using Adobe Photoshop CS3 without altering the original photo integrity.

Further examination of plants for LC‐MS/MS and RNA‐sequencing have grown as above then exposed to one of three treatments for 6 hr under gentle shaking conditions at room temperature: MES + 150 mM NaCl, MES + 20 mM H_2_O_2_, or MES buffer (3 mM MES buffer, pH 5.7) as a control. After treatment, plants were carefully patted dry to determine fresh weight, then flash frozen in liquid nitrogen and ground by mortar and pestle into a fine powder. Samples were then split to allow RNA extraction/sequencing and as well as measurement of cytokinin levels from the same samples as described below.

### 2.2 RNA extraction, library preparation, and illumina GAIIX sequencing

Three independent biological replicates were used to isolate total RNA using the Qiagen RNeasy Plant Mini‐kit according to the manufacturer's instructions. Total RNA then used for messenger RNA isolation with polyA selection and subsequent library construction with the TruSeq RNA sample preparation protocol from Illumina (San Diego, CA). Three biological replicates were sequenced and analyzed for each of the three treatment combinations. Single‐end sequencing was performed on the nine samples by the Illumina GAIIX platform, generating 186,460,653 1 × 54 bp reads. Raw sequence data is available for download at NCBI Sequence Read Archive under the BioProject ID: PRJNA476376, SRA accession: SRP150651.

### 2.3 Illumina mRNA sequence data analysis

High‐quality sequence data generated for each of the nine samples were aligned to Solanum lycopersicum genome downloaded from NCBI as performed in Gupta et al., 2013 (ftp://ftp.ncbi.nlm.nih.gov/genomes/Solanum_lycopersicum/). The associated annotation file, GFF format, was used to obtain genic information for downstream analysis. BAM alignments were generated using GSNAP (Genomic Short‐read Nucleotide Alignment Program) (version released on 2013_05_09) (Wu & Watanabe, 2005) with the following parameters; indel penalty = 2, maximum mismatches = 0.06, terminal threshold = 1,000, novel splicing = 1, local splice distance = 10,000, distant splice penalty = 1,000 and everything else set to default. Read counts were generated using NCGR's in‐house pipeline, ALPHEUS (Miller et al., 2008) as previously performed in Gupta et al., 2013;. Gene expression for each of the nine samples was computed as a measure of the total number of reads uniquely aligning to the reference genome, binned by genic coordinates (information acquired from the annotation GFF3 file). Differential gene expression analysis was performed using the R (R core Team 2013) (http://www.R-project.org/) Bioconductor package DESEQ (Anders & Huber, 2012). Raw read counts obtained were normalized to account for differences in sequencing depth and composition using methods implemented within DESEQ package. Differential expression of pairwise comparisons (combinations of the different conditions) was assessed using the negative binomial test with the Benjamani–Hochberg false discovery rate (FDR) adjustment applied for multiple testing corrections. For this study, an FDR of 0.05 was applied and any candidate that had a *p*‐adjusted value of ≤ 0.05 was considered to be significantly regulated. Full lists of gene expression are given in Supporting Information Table S2.

Generation of a tomato cytokinin‐regulated list was generated by combined lists of all RNA‐sequenced DEGs after cytokinin treatment previously performed in tomato (Gupta et al., 2013; Shi et al., 2013) and removing duplicates as shown in Supporting Information Table S5. This list of nearly 1,100 DEGs was directly compared to the lists of stress DEGs found in this study.

Gene ontology GO analysis for overrepresentation was examined using the Overrepresentation Test at the PantherDB.org webpage using default settings. GO complete categories (Molecular Function, Biological Process, and Cellular Component) were examined using a False Discovery Rate (FDR < 0.05) for each set of DEGs for salt and oxidative stress separated using distinct induced and repressed lists (Supporting Information Table S4). Additional examination of the combined cytokinin‐regulated list was performed in the same manner (Supporting Information Table S5).

### 2.4 Confirmation of RNA‐sequencing by qPCR

Five genes identified as DEG under stress conditions vs buffer control were selected to validate the RNA‐sequencing results using quantitative real‐time PCR analysis (qPCR) following a modified protocol from Shi et al., 2013;. Total RNA for each treatment was extracted as described above then used to generate cDNA for qPCR by reverse transcription using Quanta qScript cDNA supermix. qPCR was performed using PerfeCTa SYBR Green Supermix (QuantaBio, Beverly, MA) in 20 μL reactions on an Eppendorf realplex2 (Hamburg, Germany). The qPCR conditions were as follows: 15 s 95°C, 20 s 58°C, 30 s 68°C (40 cycles), followed by melt curve analysis. All qPCR reactions were performed using two biological replicates and three technical replicates. For these replicates plants were grown and treated under identical conditions as for transcriptome analysis. Fold change was calculated using the delta CT method with TIP41 as a control gene. Analysis for change was analyzed using a one‐tailed *t*‐Test and *p *<* *0.05 as a cut off for significance. Primer sequences for the genes which were verified through qPCR are presented in Supporting Information Table S3.

### 2.5 Measurements of cytokinin level

Extraction and quantification of cytokinins were performed as described previously (Svačinová et al., 2012) using the LC‐MS/MS system consisting of an ACQUITY UPLC System and Xevo TQ‐S triple quadrupole mass spectrometer (Waters). Results are presented as the average of five biological replicates ± standard deviation in pmol/g FW for samples treated and described above. Statistical examinations were made between buffer control treated and stress treatments using an ANOVA analysis with *p *<* *0.05.

### 2.6 Cytokinin‐abiotic stress senescence assay

For salt stress priming and recovery, 50‐60‐days‐old plants (MicroTom) were used to make leaf disks (1 mm diameter) and then floated in 3 mM MES buffer in cell culture plates. Full treatments were made either in buffer as noted, + salt (150 mM NaCl) or + cytokinin (5 μM BA) for the entire experiment. Pretreatment included treatment at the initiation of the experiment followed by an addition of another treatment or buffer at 48 hr indicated as a post‐treatment. Pre and post‐treatments were performed using the same levels of salt and cytokinin as in full treatments. Oxidative stress priming and recovery were conducted in a similar manner as for salt stress, except 15‐days‐old plants (M82) were used and 20 mM H_2_O_2_ was used as an oxidative stress treatment. Leaf disks were grown under standard conditions. *F*_v_/*F*_m_ measurements were performed in a manner similar to as in Zwack et al., 2016 with 9–18 leaf disks were used for all experimental treatments examined and conducted in triplicate biological replicates with analysis using Student's *t*‐test. Additional experiments performed under each stress were performed in both M82 and MicroTom cultivars with similar findings as presented in the results section (data not shown).

## 3 RESULTS

### 3.1 Examination of cytokinin regulation by salt and oxidative stress reveals distinct responses

**Figure 1:**
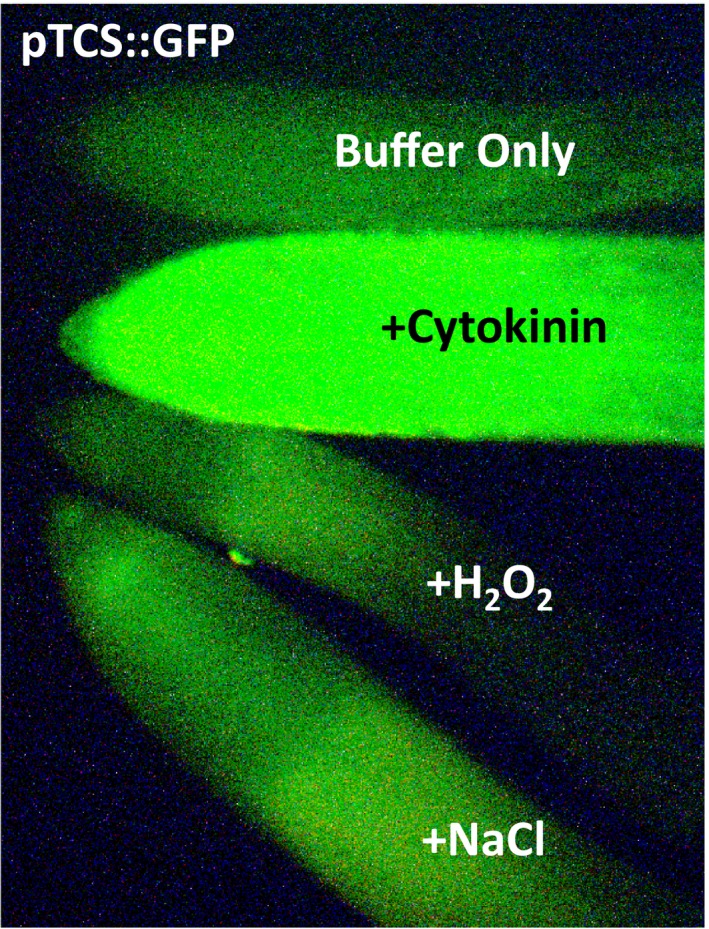
Stress interactions with a cytokinin‐responsive YFP reporter line (TCS::VENUS). Visualization of the stable cytokinin‐responsive YFP reporter line (pTCS::VENUS) in tomato roots. pTCS::VENUS was treated for 24 hr in MES buffer, Cytokinin (5 μM *trans*‐zeatin), Oxidative (20 mM H_2_O_2_) or Salt (150 mM NaCl) stress. Representative fluorescing root tips are shown. Results show a strong increase in YFP expression after cytokinin treatment as expected. Oxidative stress shows a reduction, while Salt stress shows a moderate increase in expression compared to Buffer only, indicating stress interaction with cytokinin

Cytokinins have been previously connected to various stress responses in plants. To further examine these, several approaches were taken. A simple examination of the cytokinin‐responsive reporter line pTCS::VENUS that is well‐known to indirectly indicate cytokinin levels in tomato roots (Bar et al., 2016; Zürcher et al., 2013) was examined under salt and oxidative stress treatments. We found as previously shown that cytokinin (5 μM *trans*‐zeatin) strongly induces YFP (Yellow Florescence Protein) fluorescence over a buffer only control treatment in young seedlings after 24 hr (Figure 1). There was also a smaller qualitative change in YFP fluorescence in response to stress versus buffer control: fluorescence appeared to be induced with salt (150 mM NaCl) and repressed with oxidative stress (20 mM H_2_O_2_) (Figure 1). Although these stress‐induced changes are not as dramatic as the change seen with cytokinin treatment, they do suggest that cytokinin levels might be affected by stress.

**Figure 2:**
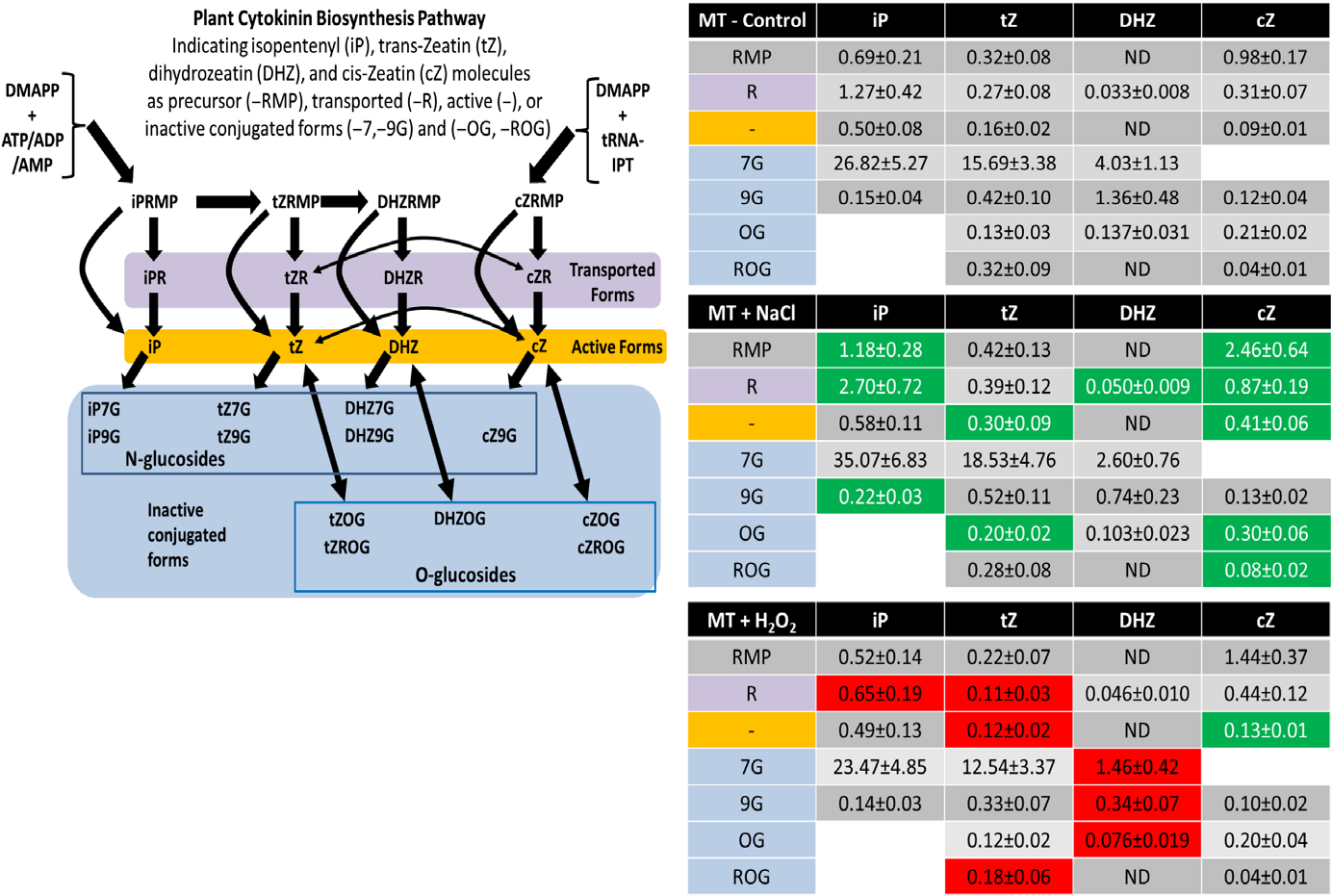
Cytokinin levels are altered by abiotic stress**.** (Left) Biosynthetic pathway for cytokinins in plants. Abbreviations in text. (Right) Specific amounts of cytokinin compounds measured by LC/MS in pmol/g FW from 10 days tomato seedlings without (Control), Salt (150 mM NaCl) or Oxidative Stress (20 mM H_2_0_2_) treatment for 6 hr (*n* = 5). Pastel colors indicate cytokinin form shown as in the biosynthetic pathway. Green/Red colors indicate significant (*p *<* *0.05) increase/decrease versus untreated control. Results show general increases in various cytokinin levels in response to salt stress, in contrast to decreasing levels seen for oxidative stresses

To directly examine cytokinin level changes in response to these stress treatments, LC‐MS/MS measurements of salt and oxidative stress‐treated seedlings (10 days) were performed. Soil grown seedlings were gently washed and placed in MES buffer with light shaking, then exposed to either salt (150 mM NaCl), oxidative stress (20 mM H_2_O_2_), or the buffer control for 6 hr. Twenty‐two different cytokinin forms were detected in this study including precursors, active, transported, and conjugated forms of isopentenyladenine (iP), *trans*‐zeatin (*tZ*), dihydrozeatin (DHZ), and *cis*‐zeatin (*c*Z). Cytokinin levels in buffer‐treated control plants show general similarities to those previously found in tomato (Žižková et al., 2015) and other eudicot plants with some variation (Sakakibara, 2006). The most abundant components are the N‐glucoside forms (iP7G, tZ7G, and DHZ7G), with the active bases iP, *t*Z, and *cZ* making up a small part of the total measured cytokinins (Figure 2). Salt treated plants showed a significant increase in 11 individual cytokinins, including the important active form *t*Z, as well as the total amount of cytokinin bases, ribosides, and nucleotide forms (Figure 2). Although there is more than a 25% increase in overall cytokinin levels compared to control, this was not significant, likely due to the lack of a significant increase in total levels of cytokinin conjugates. Oxidative stress‐treated plants showed the opposite result with significantly decreased levels of seven different cytokinin forms, including *t*Z. There is also an opposing overall 20% decrease in cytokinin levels, although not significant, again likely due to nonsignificant changes in the high levels of conjugate cytokinin forms. However, a comparison of overall cytokinin levels in plants between salt and oxidative stress treatment is significantly different (*p *<* *0.01) indicating a clear difference in cytokinin levels between these two stresses. This result is similarly true when comparing of all general classes of cytokinins to each other (bases, ribosides, nucleotides, O‐glucosides, and N‐glucosides) at *p *<* *0.05 level (Supporting Information Table S1). Interestingly, the only significantly regulated cytokinin under both stresses was *cZ*, which has previously been suggested to play a role in environmental stress response (Schäfer et al., 2015).

### 3.2 Transcriptomic effects of salt and oxidative stresses

As part of the split experimental design, plants from the same stress treatments described above were examined using RNA‐sequencing. RNA was extracted from these seedlings to produce cDNA libraries from which single‐end Illumina GAIIX RNA‐sequencing was performed. This generated a total of 186,460,653 1 × 54 bp reads from all samples, which were aligned to the *S. lycopersicum* reference genome. Further analysis resulted on average in 14.3 million uniquely aligning reads per sample from which gene expression was quantified, using the total number of reads per sample that uniquely aligned to the reference binned by gene (Table 1). Genes used for differential expression (DE) analysis were restricted to those found to be significantly regulated based on a *p*_adj_ < 0.05 (Supporting Information Table S2) as compared between stress‐treated and untreated control.

**Table 1:** Total reads from RNA‐sequencing runs

Sample	Total RNA reads	Uniquely aligned reads
MT‐Control rep1	20,105,395	12,102,357
MT‐Control rep2	8,608,669	5,648,071
MT‐Control rep3	29,611,517	17,630,048
MT + H_2_0_2_ rep1	18,838,675	14,791,987
MT + H_2_0_2_ rep2	22,658,651	17,233,910
MT + H_2_0_2_ rep3	15,577,188	12,031,139
MT + NaCl rep1	18,086,167	11,616,237
MT + NaCl rep2	21,937,106	15,517,603
MT + NaCl rep3	31,037,285	22,555,867

Note

Below is the total number of RNA‐sequencing reads from each biological replicate of as well as the number of those reads that were then uniquely aligned to the Heinz tomato reference genome. Samples are of the *Solanum lycopersicum* cv. Microtom (MT) either buffer‐treated (Control), or stress treated (+20 mM H_2_O_2_), (+150 mM NaCl) for 6 hr.

qPCR was performed to confirm the results of RNA‐sequencing on five DE genes affected by salt and oxidative stress treatments (Table 2). This comparison yielded similar expression directionality and level of regulation for genes examined indicating that the log_2_ fold change values obtained from RNA‐sequencing were accurate. In addition to confirming the overall validity of the RNA‐sequencing data, these qPCR data suggest that a number of hormone‐related genes are affected under stress conditions, including ACC synthase (ethylene), IAA‐Amido Synthetase (auxin), and His‐containing phosphotransmitter Hpt4‐like (cytokinins). Universal Stress Protein A and a sodium‐proton antiporter were also altered by stress.

**Table 2:** qPCR Confirmation of RNA‐sequencing transcriptomic results

Solyc #	Gene	Description	MT vs. MT+NaCl	MT vs. MT+H2O2
Solyc01g107400	IAA‐AS	Indole‐3‐Acetic Acid‐Amido Synthetase	3.29 ± 0.05	2.04 ± 0.02
Solyc08g081550	ACS	1‐Aminocyclopropane‐1‐Carboxylate Synthase‐like	1.96 ± 0.10	1.21 ± 0.02
Solyc06g008820	NHX1	Sodium Hydrogen Exchanger‐like	1.23 ± 0.01	1.30 ± 0.01
Solyc09g011670	USPA	Universal Stress Protein A	0.82 ± 0.01	1.28 ± 0.00
Solyc08g066350	HPT4‐like	Histidine‐Containing Phosphotransfer 4‐like	0.72 ± 0.00	1.18 ± 0.04

Note

Five genes found to be DE for at least one stress comparison to buffer‐treated samples by RNA‐sequencing were selected for further verification of expression change by qPCR. qPCR was performed using two biological replicates and three technical replicates treated in the same manner as for RNA‐sequencing experiments and normalized to TIP41 gene expression as a control. All qPCR expression changes follow RNA‐sequencing expression changes in a significant manner (*p *<* *0.05, *T*‐test) as indicated by font color (green‐induced, red‐repressed, gray‐unchanged).

**Figure 3:**
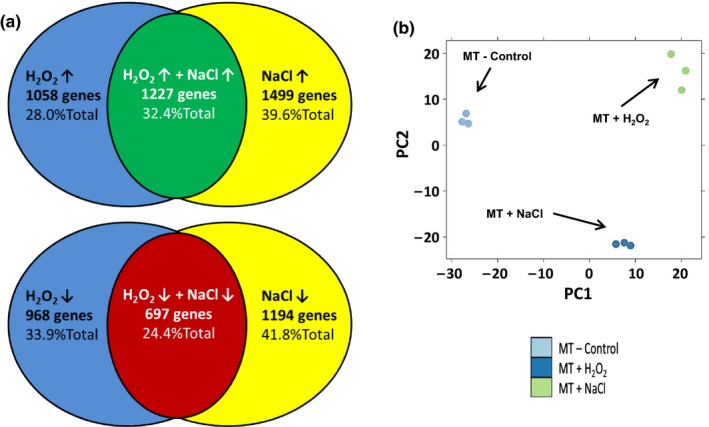
Transcriptome analysis reveals distinct patterns abiotic stress gene regulation. (a) Venn diagrams indicating the numbers of tomato genes significantly regulated by H_2_O_2_ and NaCl stress treatments: Top – induced genes, Bottom – repressed genes. While 32.4% of the induced and 24.4% of the repressed genes overlap between these stresses, the majority 67.6% of the induced and 75.6% of the repressed genes show unique stress regulation. (b) PCA analysis of each transcript replicate indicates a strong and distinct clustering of due to treatment (PC1) as well as a major component of transcript differences due to type of stress (PC2)

Overall transcriptomic changes under each stress condition were compared using the lists of significantly differentially expressed genes (DEGs) in a Venn diagram, 3,950 DEGs were identified under oxidative stress and 4,617 DEG were found under salt stress. A similar number of DEGs has been found to be regulated by other stresses in other systems (Nishiyama et al., 2011). While abiotic stresses are sometimes thought of as triggering similar gene expression changes in plants, here we see strong differences between the overall set of stress‐regulated DEGs where 71.0% of all DEGs show unique stress regulation and only a 29% overlap. A similar trend is found when the stress induced and repressed gene lists are separately compared to each other: 67.6% of induced and 75.6% of repressed DEGs show unique stress regulation (Figure 3a). In contrast, almost no induced genes from one stress are repressed by the other list: only 38 oxidative stress induced and salt repressed, and 74 salt‐induced and oxidative stress repressed. Additionally, a Principal component analysis (PCA) and variance decomposition (both as implemented in SAS JMP Genomics 5.1) of the overall, full transcriptome dataset (*n* = 9) showed distinct differences between each set of treatment samples and the buffer control (Figure 3b). Approximately 35% of the variation appears to be from treatment, with at least 20% of the variation appears as a difference between salt and oxidative stress‐treated samples (Figure 3b). Together this suggests a distinct transcript pattern of gene regulation from salt versus oxidative stress.

To additionally compare patterns of gene regulation between salt and oxidative stress, GO (Gene Ontology) enrichment analysis was performed on the lists of DEGs for each stress. As each gene list contained a large number of DEGs, lists were further divided into induced and repressed lists for each stress. Importantly, in each of these lists, response to abiotic stress was found as significantly enriched 2.0‐2.5‐fold (Table 3). Several other stress or stimulus‐response GO terms were also found as enriched (Table 3). Distinct GO terms were found as significantly enriched for each stress (Supporting Information Table S4). One such example is for ethylene‐related GO categories (biosynthetic, metabolic, and response to ethylene) that were significantly enriched for oxidative yet not for salt stress. There are also a number of GO terms that are enriched for both stresses only when comparing the induced lists (calcium ion/calmodulin binding, glucosyltransferase activity, phosphatase activity) and similarity only when comparing the repressed lists (chlorophyll binding, structural constituent of cytoskeleton). Based on changes that were seen from examination of direct and indirect measurements of cytokinins in Figures 1 and 2, it would be predicted that some cytokinin‐related category would be found as enriched from this GO analysis, although that was not the case. We believe that this is due in part to poor annotation of GO terms in descriptions of cytokinin‐related genes in tomato.

**Table 3:** Gene ontology enrichment analysis of salt and oxidative stress DE genes

	Fold enrichment	FDR		Fold enrichment	FDR
*Salt stress induced*			*Oxidative stress induced*		
Response to abiotic stimulus	2.07	5.29E‐03	Oxidation‐reduction process	1.68	1.94E‐09
Response to stress	1.53	2.80E‐04	regulation of response to stimulus	2.25	3.03E‐02
Regulation of response to stress	3.27	1.81E‐02	Response to stimulus	1.77	2.88E‐17
Regulation of response to stimulus	2.99	2.14E‐05	Response to abiotic stimulus	2.37	4.26E‐04
Response to stimulus	1.64	1.80E‐14	Response to oxygen‐containing compound	2.45	1.38E‐03
Cellular response to stimulus	1.43	1.25E‐04	Cellular response to oxidative stress	3.03	4.25E‐02
			Cellular response to stimulus	1.35	8.88E‐03
*Salt stress repressed*			Response to oxidative stress	2.15	1.41E‐02
Response to abiotic stimulus	2.25	8.95E‐03			
Response to oxidative stress	2.2	4.60E‐02	*Oxidative stress repressed*		
Response to stimulus	1.38	1.02E‐03	Response to abiotic stimulus	2.42	7.66E‐03

Note

Gene ontology GO analysis for overrepresentation was examined using an Overrepresentation Test at the PantherDB.org webpage using default settings. Stress and oxidative categories with a False Discovery Rate (FDR < 0.05) for each stress separated by induced and repressed DEGs are shown.

To more thoroughly examine the regulation of cytokinins under stress conditions, a manual examination of DEG lists was made, using search terms such as “cytokinin” and “zeatin” as well as searching by Solyc# based on previous publications of cytokinin‐related gene lists and phylogenetic comparisons to between tomato and Arabidopsis (Capua & Eshed, 2017; Sun et al., 2017). From that examination 35 cytokinin‐related DEGs could be identified as significantly regulated by stress (Table 4). These 35 genes are involved in cytokinin biosynthesis, transport, and signaling, yet only three were found with GO identifiers relating to cytokinin. Twenty‐five of these genes show a unique regulation between salt and oxidative stresses: 10 oxidative stress only, 15 salt only, and 10 both oxidative and salt regulated.

**Table 4:** Cytokinin‐related genes are altered by salt and oxidative stress treatment

Gene ID	Name/Description	FC	Function	Gene ID	Name/Description	FC	Function
*Salt induced*				*Oxidative stress induced*			
Solyc01g105360	UGT85A1‐like glucosyltransferase	22.94	Biosynthesis	Solyc04g016230	Zeatin O‐xylosyltransferase	18.30	Biosynthesis
Solyc10g079320	Zeatin O‐glucosyltransferase	15.50	Biosynthesis	Solyc12g057080	UGT85A1‐like glucosyltransferase	17.38	Biosynthesis
Solyc04g016230	Zeatin O‐xylosyltransferase	13.74	Biosynthesis	Solyc07g006800	Zeatin O‐glucosyltransferase	5.11	Biosynthesis
Solyc08g061930	Cytokinin Oxidase	6.75	Biosynthesis	Solyc04g080820	Cytokinin Oxidase (SlCKX4)	4.24	Biosynthesis
Solyc03g078490	UGT85A1‐like glucosyltransferase	6.16	Biosynthesis	Solyc10g079930	Zeatin O‐glucosyltransferase	3.82	Biosynthesis
Solyc07g006800	Zeatin O‐glucosyltransferase	5.96	Biosynthesis	Solyc12g057060	UGT85A1‐like glucosyltransferase	3.77	Biosynthesis
Solyc10g079930	Zeatin O‐glucosyltransferase	5.80	Biosynthesis	Solyc11g066670	Zeatin O‐glucosyltransferase	3.47	Biosynthesis
Solyc03g120320	F‐box kelch‐repeat KMD‐like	5.11	Signaling	Solyc03g078490	UGT85A1‐like glucosyltransferase	2.09	Biosynthesis
Solyc02g071220	Type‐A Response regulator ARR8‐like	3.45	Signaling	Solyc10g079600	Type‐A Response regulator ARR9‐like	2.00	Signaling
Solyc04g081290	LOG1‐like	3.24	Biosynthesis	Solyc01g088550	PUP11‐like Transporter	1.92	Transport
Solyc10g079600	Type‐A Response regulator ARR9‐like	2.72	Signaling	Solyc08g062820	LOG8‐Like	1.66	Biosynthesis
Solyc02g090400	Type‐B Response regulator ARR13‐like	2.64	Signaling	Solyc05g054390	Type‐B Response regulator ARR1‐like	1.49	Signaling
Solyc11g066670	Zeatin O‐glucosyltransferase	2.55	Biosynthesis				
Solyc07g005660	PUP5‐like Cytokinin Transporter	2.42	Transport				
Solyc04g074870	PUP3‐like Cytokinin Transporter	2.30	Transport				
Solyc08g062820	LOG8‐Like	1.94	Biosynthesis				
Solyc05g054390	Type‐B Response regulator ARR1‐like	1.90	Signaling				
Solyc12g057060	UGT85A1‐like glucosyltransferase	1.64	Biosynthesis				
Solyc05g015610	Cytokinin Receptor HK3	1.55	Signaling				
			
*Salt repressed*				*Oxidative stress reduced*			
Solyc08g066350	Histophosphotransfer Protein Hpt4‐like	Neg Inf	Signaling	Solyc10g079700	Type‐A Response regulator ARR9‐like	−4.41	Signaling
Solyc06g048600	Type‐A Response regulator ARR17‐like	−5.89	Signaling	Solyc12g087870	PUP3‐like Cytokinin Transporter	−2.51	Transport
Solyc01g098400	Histophosphotransfer Protein Hpt1‐like	−2.89	Signaling	Solyc05g006420	Type‐A Response regulator ARR5‐like	−2.51	Signaling
Solyc02g079330	ENT3‐like Cytokinin Transporter	−2.60	Transport	Solyc08g081960	Cytokinin Response Factor SlCRF2	−2.42	Signaling
Solyc04g016190	Zeatin O‐glucosyltransferase	−2.24	Biosynthesis	Solyc04g008110	Cytokinin Receptor HK4	−1.77	Signaling
				Solyc07g047770	Cytokinin Receptor HK2	−1.69	Signaling
				Solyc04g016190	Zeatin O‐glucosyltransferase	−1.68	Biosynthesis

Note

Genes with cytokinin‐related functions (biosynthesis (biosynthesis/metabolism), signaling, or transport) show regulation after stress treatment. Results are presented in average Fold Change (FC) compared to a buffer‐treated control of transcriptome (RNAseq) analyses of 10‐days‐old Salt (150 mM NaCl) or Oxidative (20 mM H_2_O_2_) stress treatment for 6 hours, *n* = 3 biological replicates, similar to plants with cytokinin levels measured.

### 3.3 Examination of cytokinin treatment on photosystem II efficiency under oxidative and salt stress

**Figure 4:**
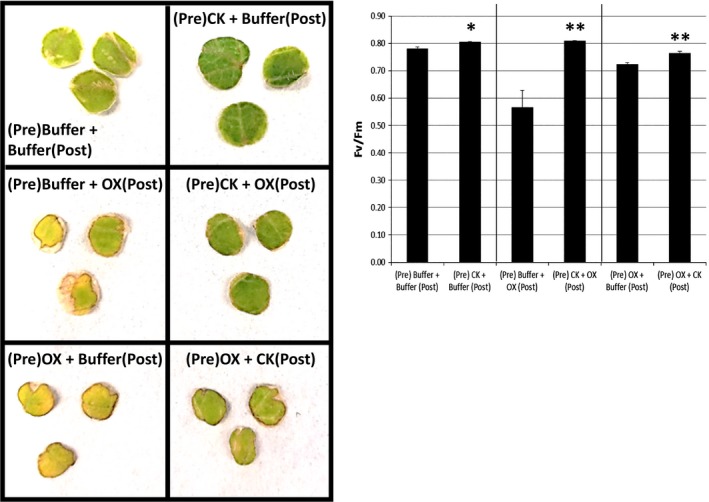
Cytokinin and oxidative stress treatment interactions. Visualization and Ave ± SE of *F*_v_/*F*_m_ of Tomato leaf disks treated with cytokinin (CK, 5 μM *trans*‐zeatin) or oxidative stress (OX, 20 mM H_2_O_2_). Initial treatment (Pre) was followed, after 48 hr by an additional treatment (Post). * indicates significance at *p *<* *0.05, ** *p *<* *0.01. Results show CK improves *F*_v_/*F*_m_, while oxidative stress decreases it. Both pre‐ and post‐treatments with CK significantly lessen oxidative stress reduction in *F*_v_/*F*_m_ levels

**Figure 5:**
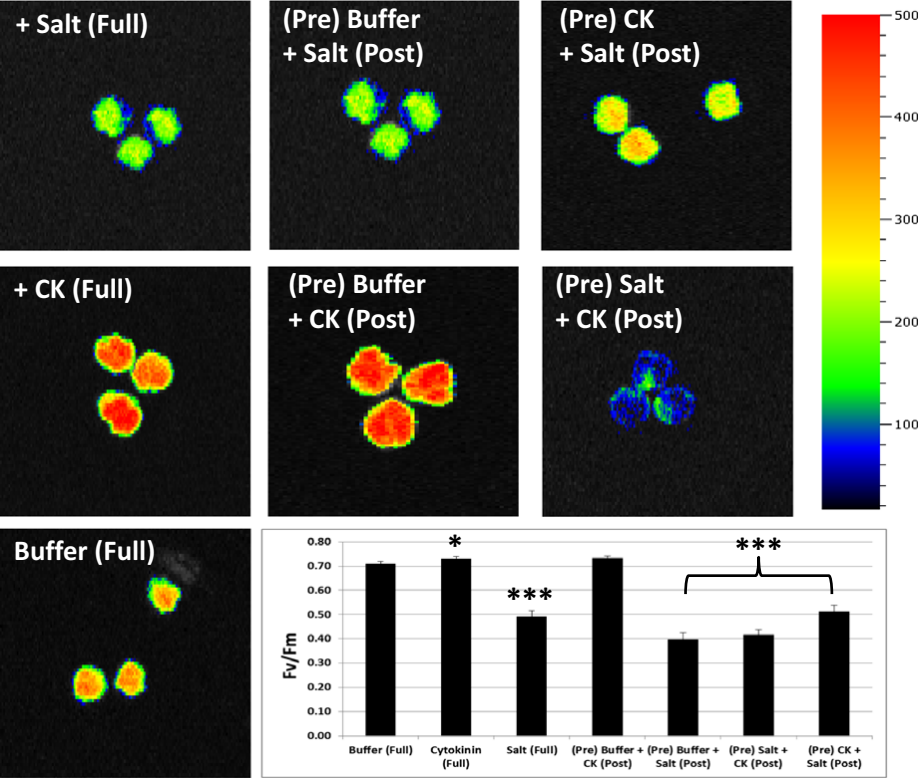
Cytokinin and salt treatment interactions. Visualization and Ave ± SE of *F*_v_/*F*_m_ of tomato leaf disks treated with cytokinin (5 μM BA) or salt (150 mM NaCl) on a relative color scale. Total treatment was for 96 hr: (Pre) pretreatments for the first 48 hr, followed by additional 48 hr treatment (Post). * indicates significance at *p *<* *0.05, *** *p *<* *0.001. Results show CK treatment improves *F*_v_/*F*_m_, while salt decreases it. Buffer pretreatments are similar to full treatment times. CK pretreatment lessens salt reduction in *F*_v_/*F*_m_, but post‐treatment cannot rescue salt treatment

Based on the findings that cytokinin levels can be affected under specific stress conditions as seen from both cytokinin measurements and transcriptomic results (Figures 1, 2, 3, Table 3), we further examined the effects of cytokinin on abiotic stress response. The classic cytokinin senescence bioassay, normally used to examine developmental senescence, was modified to examine abiotic stress responses of tomato leaf disks (Mok & Mok, 2001; Zwack, Robinson, Risley, & Rashotte, 2013). Previous work, again verified here, shows that cytokinin treatment of leaf disks reduces leaf senescence as measured using a chlorophyll fluorimeter to determine the efficiency of photosystem II or *F*_v_/*F*_m_ (Zwack et al., 2016; Figures 4 and 5). Oxidative stress (20 mM H_2_O_2_) reduces *F*_v_/*F*_m_ by 10%–30% over simple buffer treatment. However, application of exogenous cytokinin (5 μM BA) either before (pre – initial stress treatment 0 hr) or after (post 48 hr) this oxidative stress treatment significantly increases *F*_v_/*F*_m_ levels over stress treatment alone (Figure 4). This is consistent with findings here that cytokinin levels are reduced in oxidative stress‐treated plants (Figures 1 and 2), such that the exogenous application to raise that level could potentially restore plants to a normal functioning level.

Salt stress (150 mM) treatments show a similar reduction in *F*_v_/*F*_m_ to 30%–40% of buffer‐treated leaf disks (Figure 5). A cytokinin pretreatment is able to significantly reduce the drop in *F*_v_/*F*_m_ levels after a salt treatment, although salt still has an effect compared to buffer or cytokinin treatment alone (Figure 5). While a cytokinin application postsalt stress treatment was unable to increase *F*_v_/*F*_m_ levels. As these results show cytokinin levels were increased after salt stress treatments (Figure 2), it seems that further increasing cytokinin levels after stress has begun has no remediation effect and a cytokinin application has only a minor effect once the salt stress is given.

## 4 DISCUSSION

Tomato production and yield is highly dependent on healthy photosynthesizing and fully functioning plants that can resist or tolerate abiotic stress. A greater understanding of the genes that regulate salt and oxidative stress resistance and tolerance is highly valuable toward the improvement of healthy tomato plants and fruits grown under field conditions. Whereas many studies of tomato have focused on older reproductively active plants or on fruits, here, we examined stress response in young seedlings that are sensitive to both salt and oxidative effects. Although fruit production is the ultimate goal of tomato cultivation, abiotic stress in young tomato seedlings can lead to highly reduced plant growth, fruit production, and death (reviewed in Acosta‐Motos et al., 2017). As cytokinins are well known to be involved in growth and development with links to delaying senescence, this study examined connection between cytokinins and abiotic stress in tomato seedlings.

The first indication we identified of a connection between cytokinins and both salt and oxidative stress was seen in the cytokinin‐responsive transgenic reporter line in tomato: pTCS::VENUS (Capua & Eshed, 2017). Here we found changes in YFP fluorescence in response to each stress in the roots of this stable tomato pTCS::VENUS line, specifically designed to show cytokinin responsiveness (Figure 1). Salt (NaCl) treatment resulted in some increase in YFP fluorescence over buffer control treatment. Although this induction is not nearly as strong as with cytokinins, it does indicate there is an interaction between salt stress and cytokinin response. In contrast, a H_2_O_2_ oxidative stress treatment results in reduced levels of cytokinin‐responsive YFP fluorescence, suggesting that cytokinin activity is negatively regulated by oxidative stress treatment. While these findings indicate indirect cytokinin connections to each stress through activation/repression of this type‐B response regulator binding site linked to YFP, it provided a basis to conduct direct and quantifiable measurements of cytokinin levels under these same stress conditions.

Direct measurements of cytokinin levels using LC‐MS/MS of stress‐treated tomato seedlings found similar evidence of distinct regulation for each stress (Figure 2) as seen with the pTCS::VENUS reporter line over a longer exposure. Seedlings exposed to salt stress (150 mM NaCl for 6 hr) showed a significant increase in several different cytokinin forms over buffer‐treated samples, including precursors, transported, active and conjugated forms (Figure 2). Importantly the highly active form *t*Z is significantly increased to nearly 200% the level of untreated plants and levels of *cZ* are even more highly increased. Although *cZ* is known to be a less active cytokinin form it has been previously connected to salt and other stress responses (Prerostova et al., 2017; Schäfer et al., 2015). Together this shows that cytokinin levels are increased in young tomato seedlings in response to salt stress. Similar increases in cytokinin levels have been previously found in connection to salt stress (Joshi et al., 2018; Prerostova et al., 2017; Šimura et al., 2018; Veselov et al., 2017; Žižková et al., 2015). However, there are also a number of studies where decreased levels of cytokinins are seen after salt stress. Interestingly many of those studies were of developmentally older plants or specific tissues, such as fruits exposed to stress treatments of greater length, sometimes weeks in duration (Ghanem et al., 2008, 2011; Nishiyama et al., 2011). This may suggest developmental differences in response to salt or possibly a more pronounced effect of general oxidative stress effects after a lengthy salt exposure. Additionally, it has been found that plants with reduced cytokinin levels, due to decreased cytokinin synthesis or increased degradation, can have increased tolerance to abiotic stresses including salt stress (Ghanem et al., 2008, 2011; Mackova et al., 2013; Nishiyama et al., 2011; Veselov et al., 2017; Žižková et al., 2015). Taken together the results of our examinations of salt stress and cytokinins indicate a clear interaction, which may vary depending on the experimental parameters being addressed.

Oxidative stress treatment (20 mM H_2_O_2_ for 6 hr) resulted in an overall reduction in the levels several different cytokinin forms (active, transported, and conjugated) in contrast to the increases seen under salt stress (Figure 2). The active *t*Z form was found to be significantly reduced 25% after oxidative stress compared to buffer treated and a full 2.5‐fold lower when compared to levels after salt stress treatment (Figure 2). It is interesting to note that the one cytokinin form found to be at significantly increased levels was *cZ*, which was also induced by salt, although to a much higher level. It is possible that *cZ* functions in a general stress role as previously noted (Schäfer et al., 2015). As this is the first study specifically examining effects of oxidative stress treatment on cytokinin levels it is difficult to find comparable measurement data, however, a general reduction in cytokinin levels is often found in response to different stresses, as seen in Nishiyama et al., 2011. Our finding of reduced cytokinin levels after oxidative stress treatment is in line with this and we believe consistent with its role as a general stressor.

Total levels of cytokinin in plants showed an increase of about 25% after salt treatment and a reduction of about 20% after oxidative stress compared to buffer treatment (Supporting Information Table S1). Although each of these levels is not significantly different from buffer treatment, a comparison of total cytokinin levels between salt treated and oxidative stress‐treated plant is significantly different (*p *=* *0.011, *T*‐test). This supports a general finding of distinct changes in cytokinin levels for salt versus oxidative stress. While several cytokinin compounds that were measured are not affected in this snapshot of cytokinin levels after stress treatment, the prominent active cytokinin, *t*Z follows a differentially regulated pattern: significantly induced by salt and significantly reduced by oxidative stress. It is additionally important to note that stress responses to both salt and oxidative stress are known to have different dynamics in particular in relation to cytokinin levels, as reviewed in Zwack & Rashotte, 2015. Examinations of cytokinin levels and transcriptomic alterations in this study were performed at 6 hours after stress exposure to ensure initial changes in response had occurred. The generally similar findings of the pTCS reporter line at 24 hr and *F*_v_/*F*_m_ over longer periods needed to see alterations due to stress overall support the early findings, although additional work is needed to fully understand changes in cytokinin dynamics during stress response.

Transcriptomic findings similarly indicate distinct profiles of gene regulation in salt and oxidative stress‐treated seedlings. This can most easily be seen in Figure 3 from the Venn diagrams, where about 70% of DEGs have a unique salt or oxidative stress regulation pattern. A principal components analysis (PCA) of all transcriptome data also revealed three distinct groupings of results, with at least 30% of the differences explained by stress‐specific treatment (Figure 3b).

It is difficult to compare the transcriptome findings here with previous results in tomato as there have been relatively few genomewide transcriptomic studies in tomato and fewer under abiotic stress conditions. This is the first RNA‐sequencing based experiment that has been conducted in tomato on salt and oxidative stress. Previous large‐scale transcript experiments performed in tomato were often focused on older plants, aspects related to fruit, and most were conducted using early tomato microarrays, which allowed for only partial (~1/3) genome analysis (Ouyang et al., 2007; Sun et al., 2010). As such, expression of nearly 2/3 of tomato genes remained previously unexamined. Findings here do appear to generally parallel those for other plant species, with large numbers of genes being found to be stress regulated. Core sets of stress‐related genes that are affected under the salt and oxidative stress conditions are examined here, as evidenced from GO enrichment analysis (Supporting Information Table S4). One set of genes that we specifically looked for to be stress regulated were cytokinin‐related genes, although they were not identified as overrepresented from GO enrichment analysis. We believe this is largely due to incomplete annotation of the tomato genome as well as incomplete identification of cytokinin‐related genes in tomato. Despite these barriers, a manual search revealed 35 cytokinin‐related genes involved in biosynthesis/metabolism, transport, and signaling, which showed various levels of regulation under stress conditions (Table 4). Interestingly the pattern of gene regulation mirrored that found in the full DEG list, with about 70% showing distinct, nonoverlapping regulation between stresses. In all, 113 cytokinin‐related genes were identified, with 35 (31%) being found as DEG under stress conditions. A similar examination of Arabidopsis seedlings exposed to short‐term salt stress revealed 25 of 92 cytokinin‐related genes were affected (27%), compared here to 21% for salt alone in this study (Nishiyama et al., 2011). This suggests 20%–30% of cytokinin‐related genes appear to be stress regulated.

To examine this problem from another direction, we looked at how many cytokinin‐regulated genes in tomato could be connected to stress response. To do this we generated a combined list of DEGs after treatment by cytokinin in tomato, from the only two cytokinin‐based transcriptome experiments previously performed in tomato (Gupta et al., 2013; Shi et al., 2013) as shown in Supporting Information Table S5. From this list of nearly 1,100 DEGs just over a third overlap with the stress DEGs found in this study. Additionally, this combined tomato cytokinin‐regulated list was also found to be significantly over enriched for many different stress‐related terms, such as “response to abiotic stress” from a GO analysis (Supporting Information Table S5). While this is in many ways is an imperfect comparison based on a number of experimental sampling differences, it does suggest again the connection between cytokinins and abiotic stress regulation. Interesting most (70%) of the overlap between these lists is unique to salt or oxidative stress‐treated lists. Further supporting the finding that cytokinin‐stress interactions appear to occur in a stress‐specific manner.

We additionally examined if these cytokinin salt/oxidative stress‐specific patterns could be tested with a physiological parameter. Both salt and oxidative stresses are well known to be detrimental to plant growth and general photosynthetic processes, as previously detailed. One simple, nondestructive examination of photosystem II efficiency is the measurement of chlorophyll fluorescence or *F*_v_/*F*_m_ using a fluorimeter. The classic cytokinin senescence bioassay that was modified to study abiotic stress responses was used in this study (Letham, 1971; Zwack et al., 2013, 2016). Tomato leaf disks were floated in buffer in the presence or the absence of cytokinin, either before (pre) or after (post) salt (NaCl), or oxidative (H_2_O_2_) stress treatment. Found here, and as previously shown, photosystem II efficiency (*F*_v_/*F*_m_) is correlated with leaf senescence in response to the addition of cytokinin, which delays it, and stress, which promotes senescence (Figures 4 and 5; Zwack et al., 2016). Figure 4 shows this for oxidative stress and cytokinin interactions (visual images of leaf disks used for *F*_v_/*F*_m_ measurements are shown). Figure 5 shows this specifically for salt and cytokinin interactions (false‐colored images of leaf disks generated by the fluorimeter used for *F*_v_/*F*_m_ measurements are shown). Under salt stress, there is a rapid decrease in *F*_v_/*F*_m_ levels or an increase in senescence (Figure 5). Importantly a cytokinin pretreatment lessens the effect of salt‐induced reduction of *F*_v_/*F*_m_ levels, indicating there is an interaction between cytokinins and salt stress, potentially involved in tolerance (Figure 5). However, the addition of cytokinin postsalt treatment shows no positive effect to lessen senescence. Oxidative stress causes a similar increase in senescence that can be lessened by the addition of a cytokinin pretreatment (Figure 4). Interestingly, after oxidative stress treatment has begun a cytokinin post‐treatment is able to reduce senescence, which does not occur with salt treatment (Figure 4).

Overall we present the first examination of both cytokinin levels and changes in whole genome transcript levels to salt and oxidative stress in tomato. While we find some overlap between these two stresses, there are many distinct, stress‐specific effects. Levels of several different cytokinin forms, including the active form *t*Z are increased after salt treatment and reduced after oxidative stress treatment. Quite distinct patterns of DEG regulation were similarly found after the same salt and oxidative stress treatments. Interestingly, a number of cytokinin‐related genes were also found to be regulated in salt versus oxidative stress‐dependent manners. Additional examination of cytokinin treatments during leaf abiotic stress senescence assays measuring photosystem II efficiency further supports the finding that cytokinins are connected to stress responses in a distinct manner for salt and oxidative stresses.

## AUTHOR CONTRIBUTIONS

E.A.K. and A.M.R. designed research; E.A.K., H.T.H., T.R., L.P, A.S., F.S., O.N., and A.M.R performed research and analyzed data; E.A.K. and A.M.R. wrote the paper.

## References

[pld371-bib-0001] Acosta‐Motos, J. R. , Ortuño, M. F. , Bernal‐Vicente, A. , Diaz‐Vivancos, P. , Sanchez‐Blanco, M. J. , & Hernandez, J. A. (2017). Plant responses to salt stress: Adaptive mechanisms. Agronomy, 23, 18 10.3390/agronomy7010018

[pld371-bib-0002] Anders, S. , & Huber, W. (2012). Differential Expression of RNA‐Seq Data at the Gene Level–the DESeq Package. Heidelberg, Germany: European Molecular Biology Laboratory (EMBL).

[pld371-bib-0003] Apel, K. , & Hirt, H. (2004). Reactive oxygen species: Metabolism, oxidative stress, and signal transduction. Annual Review of Plant Biology, 55, 373–399. 10.1146/annurev.arplant.55.031903.141701 15377225

[pld371-bib-0004] Bar, M. , Israeli, A. , Levy, M. , Gera, H. B. , Jiménez‐Gómez, J. , Kouril, S. , … Ori, N. (2016). CLAUSA is a MYB transcription factor that promotes leaf differentiation by attenuating cytokinin signaling. The Plant Cell, 28, 1602–1615. 10.1105/tpc.16.00211 27385816 PMC4981134

[pld371-bib-0005] Bose, J. , Rodrigo‐Moreno, A. , & Shabala, S. (2014). ROS homeostasis in halophytes in the context of salinity stress tolerance. Journal of Experimental Botany, 65, 1241–1257. 10.1093/jxb/ert430 24368505

[pld371-bib-0006] Capua, Y. , & Eshed, Y. (2017). Coordination of auxin‐triggered leaf initiation by tomato LEAFLESS. PNAS, 114, 3246–3251. 10.1073/pnas.1617146114 28270611 PMC5373412

[pld371-bib-0007] Davies, P. J. (2004). Plant Hormones: Biosynthesis, Signal Transduction, Action!. Dordrecht, The Netherlands: Kluwer Academic Publishers.

[pld371-bib-0008] Duque, A. S. , deAlmeida, A. M. , da Silva, A. B. , da Silva, J. M. , Farinha, A. P. , Santos, D. , … deSousa Araújo, S. (2013). Abiotic stress responses in plants: Unraveling the complexity of genes and networks to survive In Vahdati K. (Ed.), Abiotic Stress ‐ Plant Responses and Applications in Agriculture. London, UK: InTech. ISBN: 978‐953‐51‐1024‐8.

[pld371-bib-0009] Ghanem, M. E. , Albacete, A. , Martínez‐Andújar, C. , Acosta, M. , Romero‐Aranda, R. , Dodd, I. C. , … Pérez‐Alfocea, F. (2008). Hormonal changes during salinity‐induced leaf senescence in tomato (Solanum lycopersicum L.). Journal of Experimental Botany, 59, 3039–3050. 10.1093/jxb/ern153 18573798 PMC2504355

[pld371-bib-0010] Ghanem, M. E. , Albacete, A. , Smigocki, A. C. , Frébort, I. , Pospíšilová, H. , Martínez‐Andújar, C. , … Pérez‐Alfocea, F. (2011). Root‐synthesized cytokinins improve shoot growth and fruit yield in salinized tomato (Solanum lycopersicum L.) plants. Journal of Experimental Botany, 62, 125–140. 10.1093/jxb/erq266 20959628 PMC2993914

[pld371-bib-0011] Gill, S. S. , & Tuteja, N. (2010). Reactive oxygen species and antioxidant machinery in abiotic stress tolerance in crop plants. Plant Physiology and Biochemistry, 48, 909–930. 10.1016/j.plaphy.2010.08.016 20870416

[pld371-bib-0012] Gupta, S. , Shi, X. , Lindquist, I. E. , Devitt, N. P. , Mudge, J. , & Rashotte, A. M. (2013). Transcriptome profiling of cytokinin and auxin regulation in tomato root. Journal of Experimental Botany, 64, 695–704. 10.1093/jxb/ers365 23307920 PMC3542057

[pld371-bib-0013] Ismail, A. M. , & Horie, T. (2017). Genomics, physiology, and molecular breeding approaches for improving salt tolerance. Annual Review of Plant Biology, 68, 1 10.1146/annurev-arplant-042916-040936 28226230

[pld371-bib-0014] Joshi, R. , Sahoo, K. K. , Tripathi, A. K. , Kumar, R. , Gupta, B. K. , Pareek, A. , & Singla‐Pareek, S. L. (2018). Knockdown of an inflorescence meristem‐specific cytokinin oxidase‐OsCKX2 in rice reduces yield penalty under salinity stress condition. Plant, Cell and Environment, 41, 936–946. 10.1111/pce.12947 28337744

[pld371-bib-0015] Letham, D. S. (1971). Regulators of cell division in plant tissues XII. A cytokinin bioassay using excised radish cotyledons. Physiologia Plantarum, 25, 391–396. 10.1111/j.1399-3054.1971.tb01462.x

[pld371-bib-0016] Mackova, H. , Hronkova, M. , Dobra, J. , Tureckova, V. , Novak, O. , Lubovska, Z. , … Vankova, R. (2013). Enhanced drought and heat stress tolerance of tobacco plants with ectopically enhanced cytokinin oxidase/dehydrogenase gene expression. Journal of Experimental Botany, 64, 2805–2815. 10.1093/jxb/ert131 23669573 PMC3741687

[pld371-bib-0017] Miller, N. A. , Kingsmore, S. F. , Farmer, A. , Langley, R. J. , Mudge, J. , Crow, J. A. , … May, G. D. (2008). Management of high‐throughput DNA sequencing projects: Alpheus. Journal of Computer Science and Systems Biology, 1, 132. 20151039 10.4172/jcsb.1000013 PMC2819532

[pld371-bib-0018] Mittler, R. (2002). Oxidative stress, antioxidants and stress tolerance. Trends in Plant Science, 7, 405–410. 10.1016/S1360-1385(02)02312-9 12234732

[pld371-bib-0019] Mittler, R. , Finka, A. , & Goloubinoff, P. (2012). How do plants feel the heat? Trends in Biochemical Sciences, 37, 118–125. 10.1016/j.tibs.2011.11.007 22236506

[pld371-bib-0020] Mittler, R. , Vanderauwera, S. , Gollery, M. , & Van Breusegem, F. (2004). Reactive oxygen gene network of plants. Trends in Plant Science, 9, 490–498. 10.1016/j.tplants.2004.08.009 15465684

[pld371-bib-0021] Mittova, V. , Tal, M. , Volokita, M. , & Guy, M. (2003). Up‐regulation of the leaf mitochondrial and peroxisomal antioxidative systems in response to salt‐induced oxidative stress in the wild salt‐tolerant tomato species Lycopersicon pennellii. Plant, Cell and Environment, 26, 845–856. 10.1046/j.1365-3040.2003.01016.x 12803612

[pld371-bib-0022] Mok, D. W. , & Mok, M. C. (2001). Cytokinin metabolism and action. Annual Review of Plant Physiology and Plant Molecular Biology, 89, 89–118. 10.1146/annurev.arplant.52.1.89 11337393

[pld371-bib-0601] Muller, B. (2011). Generic signal‐specific responses: Cytokinin and context‐dependent cellular responses. Journal of Experimental Botany, 62, 3273‐3288. 21212299 10.1093/jxb/erq420

[pld371-bib-0023] Nishiyama, R. , Watanabe, Y. , Fujita, Y. , Le, D. T. , Kojima, M. , Werner, T. , … Tran, L.‐S. P. (2011). Analysis of cytokinin mutants and regulation of cytokinin metabolic genes reveals important regulatory roles of cytokinins in drought, salt and abscisic acid responses, and abscisic acid biosynthesis. The Plant Cell, 23, 2169–2183. 10.1105/tpc.111.087395 21719693 PMC3160038

[pld371-bib-0024] Ouyang, B. , Yang, T. , Li, H. , Zhang, L. , Zhang, Y. , Zhang, J. , … Ye, Z. (2007). Identification of early salt stress response genes in tomato root by suppression subtractive hybridization and microarray analysis. Journal of Experimental Botany, 58, 507–520. 10.1093/jxb/erl258 17210988

[pld371-bib-0025] Pandey, S. K. , Nookaraju, A. , Upadhyaya, C. P. , Gururani, M. A. , Venkatesh, J. , Kim, D.‐H. , & Park, S. W. (2011). An update on biotechnological approaches for improving abiotic stress tolerance in tomato. Crop Science, 51, 2303–2324. 10.2135/cropsci2010.10.0579

[pld371-bib-0026] Peleg, Z. , & Blumwald, E. (2011). Hormone balance and abiotic stress tolerance in crop plants. Current Opinion in Plant Biology, 14, 290–295. 10.1016/j.pbi.2011.02.001 21377404

[pld371-bib-0027] Pineda, B. , Garcia‐Abellan, J. O. , Anton, T. , Perez, F. , Moyano, E. , Sogo, B. G. , … Atares, A. (2012). Tomato: Genomic approaches for salt and drought stress tolerance In Tuteja N., Gill S. S., Tiburcio A. F. & Tuteja R. (Eds.), Improving Crop Resistance to Abiotic Stress. Singapore: Wiley‐VCH.

[pld371-bib-0028] Prerostova, S. , Dobrev, P. I. , Gaudinova, A. , Hosek, P. , Soudek, P. , Knirsch, V. , & Vankova, R. (2017). Hormonal dynamics during salt stress responses of salt‐sensitive Arabidopsis thaliana and salt‐tolerant *Thellungiella salsuginea* . Plant Science, 264, 188–198. 10.1016/j.plantsci.2017.07.020 28969799

[pld371-bib-0029] Qin, F. , Shinozaki, K. , & Yamaguchi‐Shinozaki, K. (2011). Achievements and challenges in understanding plant abiotic stress responses and tolerance. Plant and Cell Physiology, 52, 1569–1582. 10.1093/pcp/pcr106 21828105

[pld371-bib-0030] R Core Team (2013). R: A Language and Environment for Statistical, Computing. Vienna, Austria: R Foundation for Statistical Computing.

[pld371-bib-0031] Ramireddy, E. , Chang, L. , & Schmülling, T. (2014). Cytokinin as a mediator for regulating root system architecture in response to environmental cues. Plant Signaling and Behavior, 9, 5021–5032. 10.4161/psb.27771 PMC4091237 24509549

[pld371-bib-0032] Sakakibara, H. (2006). Cytokinins: Activity, biosynthesis, and translocation. Annual Review of Plant Biology, 57, 431–449. 10.1146/annurev.arplant.57.032905.105231 16669769

[pld371-bib-0033] Saxena, I. , Srikanth, S. , & Chen, Z. (2016). Cross talk between H_2_O_2_ and interacting signal molecules under plant stress response. Frontiers in Plant Science, 7, 570 10.3389/fpls.2016.00570 27200043 PMC4848386

[pld371-bib-0034] Schäfer, M. , Brütting, C. , Meza‐Canales, I. D. , Großkinsky, D. K. , Vankova, R. , Baldwin, I. T. , & Meldau, S. (2015). The role of cis‐zeatin‐type cytokinins in plant growth regulation and mediating responses to environmental interactions. Journal of Experimental Botany, 66, 4873–4884. 10.1093/jxb/erv214 25998904 PMC5147713

[pld371-bib-0035] Shi, X. , Gupta, S. , Lindquist, I. E. , Cameron, C. T. , Mudge, J. , & Rashotte, A. M. (2013). Transcriptome analysis of cytokinin response in tomato leaves. PLoS ONE, 8, e55090 10.1371/journal.pone.0055090 23372818 PMC3555872

[pld371-bib-0036] Šimura, J. , Antoniadi, I. , Široká, J. , Tarkowska, D. , Strnad, M. , Ljung, K. , & Novak, O. (2018). Plant hormonomics: Multiple phytohormone profiling by targeted metabolomics. Plant Physiology, 10.1104/pp.18.00293 10.1104/pp.18.00293 PMC6001343 29703867

[pld371-bib-0037] Singh, M. , Singh, A. , Prasad, S. M. , & Singh, R. K. (2017). Regulation of plants metabolism in response to salt stress: An omics approach. Acta Physiologiae Plantarum, 39, 48 10.1007/s11738-016-2345-x

[pld371-bib-0038] Sun, Y. , Ji, K. , Liang, B. , Du, Y. , Jiang, L. , Wang, J. , … Wang, H. (2017). Suppressing ABA uridine diphosphate glucosyltransferase (SlUGT75C1) alters fruit ripening and the stress response in tomato. The Plant Journal, 91, 574–589. 10.1111/tpj.13588 28482127

[pld371-bib-0039] Sun, W. , Xu, X. , Zhu, H. , Liu, A. , Liu, L. , Li, J. , & Hua, X. (2010). Comparative transcriptomic profiling of a salt‐tolerant wild tomato species and a salt‐sensitive tomato cultivar. Plant and Cell Physiology, 51, 997–1006. 10.1093/pcp/pcq056 20410049

[pld371-bib-0040] Svačinová, J. , Novák, O. , Lenka Plačková, L. , Lenobel, R. , Holík, J. , Strnad, M. , & Doležal, K. (2012). A new approach for cytokinin isolation from Arabidopsis tissues using miniaturized purification: Pipette tip solid‐phase extraction. Plant Methods, 8, 17. 22594941 10.1186/1746-4811-8-17 PMC3492005

[pld371-bib-0041] To, J. P. C. , & Kieber, J. J. (2008). Cytokinin signaling: Two‐components and more. Trends in Plant Science, 13, 85–92. 10.1016/j.tplants.2007.11.005 18262459

[pld371-bib-0042] Veselov, D. S. , Kudoyarova, G. R. , Kudryakova, N. V. , & Kusnetsov, V. V. (2017). Role of cytokinins in stress resistance of plants. Russian Journal of Plant Physiology, 64, 15–27. 10.1134/S1021443717010162

[pld371-bib-0043] Wilkinson, S. , Kudoyarova, G. R. , Veselov, D. S. , Arkhipova, T. N. , & Davies, W. J. (2012). Plant hormone interactions: Innovative targets for crop breeding and management. Journal of Experimental Botany, 63, 3499–3509. 10.1093/jxb/ers148 22641615

[pld371-bib-0044] Wu, T. D. , & Watanabe, C. K. (2005). GMAP: A genomic mapping and alignment program for mRNA and EST sequences. Bioinformatics, 21(9), 1859–1875. 10.1093/bioinformatics/bti310 15728110

[pld371-bib-0045] Žižková, E. , Dobrev, P. I. , Muhovski, Y. , Hošek, P. , Hoyerová, K. , Haisel, D. , … Hichri, I. (2015). Tomato (Solanum lycopersicum L.) SlIPT3 and SlIPT4 isopentenyltransferases mediate salt stress response in tomato. BMC Plant Biology, 15, 85. 25888402 10.1186/s12870-015-0415-7 PMC4404076

[pld371-bib-0046] Zürcher, E. , Tavor‐Deslex, D. , Lituiev, D. , Enkerli, K. , Tarr, P. T. , & Müller, B. (2013). A robust and sensitive synthetic sensor to monitor the transcriptional output of the cytokinin signaling network in planta. Plant Physiology, 161, 1066–1075. 10.1104/pp.112.211763 23355633 PMC3585579

[pld371-bib-0047] Zwack, P. J. , De Clercq, I. , Howton, T. C. , Hallmark, H. T. , Hurny, A. , Keshishian, E. A. , … Rashotte, A. M. (2016). Cytokinin response factor 6 represses cytokinin‐associated genes during oxidative stress. Plant Physiology, 172, 1249–1258. 27550996 10.1104/pp.16.00415 PMC5047073

[pld371-bib-0048] Zwack, P. J. , & Rashotte, A. M. (2015). Interactions between cytokinin signalling and abiotic stress responses. Journal of Experimental Botany, 66, 4863–4871. 10.1093/jxb/erv172 25911740

[pld371-bib-0049] Zwack, P. J. , Robinson, B. R. , Risley, M. G. , & Rashotte, A. M. (2013). Cytokinin response factor 6 negatively regulates leaf senescence and is induced in response to cytokinin and numerous abiotic stresses. Plant and Cell Physiology, 54, 971–981. 10.1093/pcp/pct049 23539244

