# Supplementary material for: Salt and oxidative stresses uniquely regulate tomato cytokinin levels and transcriptomic response
Source: Plant Direct. 2018 Jul 19;2(7):e00071. doi: 10.1002/pld3.71 (PMC6508850; doi:10.1002/pld3.71)
Supplement: Supplementary file 6 [file PLD3-2-e00071-s006.pdf]

Response to reviewer's comments.

We are appreciative of the good comments and suggestions by the reviewers and editor and have endeavored to make those improvement to produce a better manuscript. Below are the response to specific reviewer comments. Attached are files with track changes and without showing these improvements Figure 1 and 4 have been revised only relating to text in their legends.

Reviewer 1

Point 1. We appreciate the suggestion for better micrographs be presented however, this image is meant to be largely qualitative and as such will side with the editor on the need not to replace it.

Point 2. We have revised this rationale statement to better reflect the focus of the presented results as the reviewer has suggested on the effects of cytokinin on stress response.

Point 3. The sentence regarding examination for several days has been removed from the figure legend from Fig 4 such that now specific sampling conditions are listed. Additional text has been added to the methods indicated that stress experiments were performed in each cultivar background with similar results.

Point 4. The primary comparisons of DEGs that are presented in the manuscript are Up vs Up lists and Down vs Down lists as these reflect the major findings of the results. We have made the additional comparisons Up vs Down and find almost no DEG overlap only 38 oxidative stress induced and salt repressed, and 74 salt induced and oxidative stress repressed. Text noting this has been added to the results. A GO enrichment analysis of these lists found no significant terms. The transcriptional changes found here seem to be either induced or repressed in one stress and unaffected by the other stress.

Additional minor points have been corrected in the text. The description of the Hpt4-like proteins are as given in the gene annotation, similar to the other genes in Table 4.

Reviewer 2.

The reviewer is correct that a potential role of cis-Zeatin changes in our finding were somewhat overlooked in the original submission. We have added text to indicate the potential importance of this cytokinin here and its potential role in each of the stresses examined here including the citation of references supporting these findings.

The reviewer is also correct that there are known differences of cytokinin levels that can occur over a dynamic range in response to the same stress. We have added text to the discussion to indicate that while the results presented here across several time points generally support findings of cytokinin increases in response to salt and decreases in response to oxidative stress future studies should further analyze this dynamic range in more detail. We have also added text to indicate why timing of experiments was conducted. Reporter line differences can be seen at 6h similar to that shown at 24h in Figure 1. However, these difference become more apparent by 24h when the image was taken. The 6 hour time point for cytokinin measurements and transcriptomics was selected to ensure appreciable changes had occurred after the initiation of each stress. Studies measuring Fv/Fm over longer times (48h) are needed to see changes in response to each stress, in many ways similar to what is needed for

cytokinin senescence bioassays. Actually, we have conducted Fv/Fm studies over much longer timeframes and see similar results to what is presented here.

We have added a note in to text that genes noted as biosynthesis could be (biosynthesis/metabolism)

Minor comments. We have addressed these comments through the rewording of text to appropriately address these points, such as we added the “in tomato” to the statement in the abstract regarding photosystem II efficiency. The BioProject ID at NCBI for the RNAseq data has been added.

We have additionally made corrections to the text and figure legend regarding the pTCS reporter line, which is a VENUS line not GFP line as the original submission indicated. No changes in results or findings are impacted by this as it was corrected imaged using a YFP filter using the same equipment, just incorrected notes as GFP. This was simply a typo error as we have a pTCSn:GFP line in Arabidopsis.
